# Gene expression responses in male fathead minnows exposed to binary mixtures of an estrogen and antiestrogen

**DOI:** 10.1186/1471-2164-10-308

**Published:** 2009-07-13

**Authors:** Natàlia Garcia-Reyero, Kevin J Kroll, Li Liu, Edward F Orlando, Karen H Watanabe, María S Sepúlveda, Daniel L Villeneuve, Edward J Perkins, Gerald T Ankley, Nancy D Denslow

**Affiliations:** 1 Department of Physiological Sciences and Center for Environmental and Human Toxicology, University of Florida, Gainesville, FL 32611, USA; 2 ICBR, University of Florida, Gainesville, FL 32611, USA; 3 Department of Animal & Avian Sciences, University of Maryland, College Park, MD 20742, USA; 4 Division of Environmental and Biomolecular Systems, Oregon Health & Science University, West Campus, Beaverton, OR, 97006, USA; 5 Department of Forestry & Natural Resources, Purdue University, Lafayette, IN, 47907, USA; 6 U.S. Environmental Protection Agency, ORD, NHEERL, MED, Duluth, MN, 55804, USA; 7 Environmental Laboratory, US Army Engineer Research and Development Center, Vicksburg, MS, 39180, USA; 8 Current address: Department of Chemistry, Jackson State University, Jackson, MS 39217, USA

## Abstract

**Background:**

Aquatic organisms are continuously exposed to complex mixtures of chemicals, many of which can interfere with their endocrine system, resulting in impaired reproduction, development or survival, among others. In order to analyze the effects and mechanisms of action of estrogen/anti-estrogen mixtures, we exposed male fathead minnows (*Pimephales promelas*) for 48 hours via the water to 2, 5, 10, and 50 ng 17α-ethinylestradiol (EE_2_)/L, 100 ng ZM 189,154/L (a potent antiestrogen known to block activity of estrogen receptors) or mixtures of 5 or 50 ng EE_2_/L with 100 ng ZM 189,154/L. We analyzed gene expression changes in the gonad, as well as hormone and vitellogenin plasma levels.

**Results:**

Steroidogenesis was down-regulated by EE_2 _as reflected by the reduced plasma levels of testosterone in the exposed fish and down-regulation of genes in the steroidogenic pathway. Microarray analysis of testis of fathead minnows treated with 5 ng EE_2_/L or with the mixture of 5 ng EE_2_/L and 100 ng ZM 189,154/L indicated that some of the genes whose expression was changed by EE_2 _were blocked by ZM 189,154, while others were either not blocked or enhanced by the mixture, generating two distinct expression patterns. Gene ontology and pathway analysis programs were used to determine categories of genes for each expression pattern.

**Conclusion:**

Our results suggest that response to estrogens occurs via multiple mechanisms, including canonical binding to soluble estrogen receptors, membrane estrogen receptors, and other mechanisms that are not blocked by pure antiestrogens.

## Background

Worldwide, aquatic organisms are exposed to mixtures of chemicals (e.g., pharmaceuticals, pesticides, and industrial chemicals), which enter the environment through wastewaters as well as other sources. Many of these chemicals are capable of interfering with endocrine signaling via a complex array of biomolecules (e.g., hormones) to regulate processes such as reproduction and metabolism. These endocrine disrupting chemicals (EDCs) alter signaling through a variety of mechanisms including binding to soluble sex hormone receptors or membrane receptors and acting as agonists or antagonists, or by inhibiting/inducing enzymes and proteins, which produce naturally occurring steroid hormones. Compared to other chemical pollutants, EDCs are likely to have effects at relatively low concentrations [1].

Of the EDCs, xenoestrogens have been the most studied because estrogenic effects have been observed in field studies of fish and wildlife populations [2-4]. In oviparous animals such as fish, a sensitive and robust biomarker (i.e. vitellogenin, VTG) exists for evaluating exposure to xenoestrogens. Early studies of sewage treatment effluents attributed the feminization of fish to exposure to mixtures of natural (e.g., estrone and 17β-estradiol, E_2_) and synthetic (e.g., 17α-ethinylestradiol, EE_2_) estrogens [1,5]. One of the most potent estrogens known is EE_2_, a pharmaceutical that is one of the active ingredients in contraceptives. Indeed, EE_2 _has been shown to be up to 27 times more potent than E_2 _[6]. In the United States, EE_2 _use is estimated at 170 kg/yr [7]; and in the United Kingdom, its use is roughly 26 kg/yr [8]. Measured EE_2 _surface water concentrations in the United States, United Kingdom, The Netherlands, and Germany range from 0.5 to 15 ng/L [7], and it has been frequently measured in United States streams [9].

In laboratory studies, exposures of fish to environmentally relevant EE_2 _concentrations cause a variety of effects that include testis-ova (the appearance of both sperm and egg follicles in the testis), increased plasma VTG concentrations, reduced gonad size, and altered sex ratios. Studies have used exposure durations of various lengths, including short (≤ 7 days of exposure), intermediate (7 to 28 days exposure), and long (> 28 days) term. In female fish, environmentally relevant EE_2 _exposures can increase plasma VTG concentrations [10-12] and decrease egg production [13] in long-term studies, but seem to have little or no effect on fecundity for intermediate length exposures [10,12]. In some studies, long-term exposure to EE_2 _completely inhibits spawning in fish [11,14].

Long-term EE_2 _exposure of embryos has been shown to disrupt sexual differentiation of male fish. Fathead minnow (FHM, *Pimephales promelas*) embryos continuously exposed to EE_2 _concentrations as low as 4 ng/L did not clearly sexually differentiate at 176 days post-fertilization [12]. Similarly, continuous exposure of zebrafish (*Danio rerio*) embryos to EE_2 _concentrations as low as 3 ng/L resulted in all fish having ovaries [11]. EE_2 _also reduced gonad size and circulating testosterone (T) levels [15], increased VTG [11,12,16], and arrested the developmental transition of the gonads of genetically male zebrafish [11]. The steroid also can cause hepatotoxicity, nephrotoxicity and gonadotoxicity [17]. Overall, studies to date suggest that exposure to EE_2 _elicits adverse effects on fish reproduction primarily through the feminization of male fish, and in females through cessation of spawning. These findings have alerted scientists and environmental regulators to the potential for severe adverse effects on aquatic populations [18], and, potentially, aquatic ecosystems [19]. The current research was conducted to provide a better understanding of the mechanistic basis for effects of estrogenic chemicals in fish.

Effects on gene expression have been investigated with short- and intermediate-term exposures to EE_2 _[20-22] in order to discover gene expression profiles indicative of potential adverse effects. In addition to affecting gene expression through soluble nuclear hormone receptors, it is now clear that sex hormones can also bind directly to membrane receptors and enact immediate changes in signaling via non-genomic pathways [23,24]. Specific sex hormone receptors in membranes have been identified in fish testis and ovaries for E_2 _[25,26], T [27] and progestins [28]. It is difficult to distinguish gene transcription regulation through classical receptor-dependent mechanisms, where estrogen receptor homo- and heterodimers bind to estrogen receptor elements in promoters, from action due to binding of estrogen receptors (ERs) to other transcription factors that activate through Sp1 (stimulatory protein 1) or AP-1 (activating protein 1) binding sites or that activate signaling cascades that start at the membrane. ZM189,154 (ZM) was produced by Astra-Zeneca (Alderly Park, Cheshire, UK) and there are reports that it functions as a "pure" antiestrogen in mammals [29] and in fish [30,31], meaning that it will bind to and inhibit activation of the ERs in all tissues. But even pure antiestrogens appear to fail in this regard with some genes that are regulated by E_2 _[32,33]. ICI 182,780, the most studied pure antiestrogen, can bind to membrane receptors of GnRH-producing GT1-7 cells and displace binding of E_2 _coupled to bovine serum albumin [34], suggesting that its binding to membrane receptors is inhibited, but it is not clear if this influences all E_2 _membrane activity [32]. The Atlantic croaker G protein-coupled receptor 30 has been shown to function as a membrane-bound estrogen receptor and its function is agonized by ICI 182,780 [35]. Other E_2 _activated pathways may not be inhibited by ICI 182,780, as has been shown for E_2_-stimulated gene regulation through an SP1 site [33]. ZM interactions with membrane receptors have not been studied.

Unlike mammalian species, as many as three to four different ERs have been identified in teleost fish [31,36-38] making evaluation of gene regulation by different ER isotypes even more challenging to understand than in mammalian systems. Using *in vitro *transfection experiments for largemouth bass (*Micropterus salmoides*) ERs, we have determined that ZM is equally effective at antagonizing the three soluble receptors [31].

A few studies have investigated the effects of estrogenic mixtures on fish [20,39,40] and the binary mixture of E_2 _with tamoxifen and letrizole, two antiestrogens [41]. However, no studies in fish have investigated the effects of a mixture of EE2 with the potent anti-estrogen, ZM. In this study, the objective was to determine changes in steroidogenesis and in gene expression profiles associated with different exposures by exposing adult male FHM to aqueous doses of EE_2_, (2, 5, 10 and 50 ng/L); to the pure antiestrogen, ZM (100 ng/L); and to mixtures of EE_2 _and ZM. The hypothesis we tested was that ZM in the mixture would block the action of EE_2 _on soluble ERs in the FHM gonad and effectively block gene expression changes observed with EE_2 _alone.

## Results

### Water Chemistry

Two distinct experiments were performed. In Exp 1, FHM were treated with three concentrations of EE_2 _(2, 10 and 50 ng EE_2_/L), 100 ng ZM/L or a mixture of 50 ng EE_2 _with 100 ng ZM/L. In Exp 2, FHM were treated with vehicle, 5 ng EE_2_/L or with a mixture of 5 ng EE_2 _with 100 ng ZM/L. Water concentrations of EE_2 _alone and in the mixture were close to target values but decreased after 24 h when they were again renewed to target concentrations (Table 1). Actual concentrations of ZM were not measured.

**Table 1:** Chemical analysis of water exposures.

	***EE2 Conc, ng/L^a^***			
	***Spike***	***SE^b^***	***24 h Post spike^c^***	***SE***

**Experiment 1**				
TEG^d^	BDL^e^		BDL	
EE2-2	1.16	0.12	0.23	0.03
EE2-10	9.02	0.59	0.13	0.05
EE2-50	57.92	0.61	6.84	1.43
Mixture EE2-50/ZM-100	60.36	1.82	24.3	2.07
				
**Experiment 2**				
TEG	BDL		BDL	
EE2-5	2.98	0.17	2.78	0.41
Mixture EE2-5/ZM-100	3.50	0.30	0.73	0.1

a, Detection limit for the ELISA in 50 ng/L

b, SE, Standard error

c, Concentration of EE2 in tank at the end of 24 h and before exposure solutions were replaced

d, TEG, triethylene glycol; EE2, 17α-ethinylestradiol; ZM, ZM 189,154

e, BDL, below detection limit

### Biological responses

**Figure 1:**
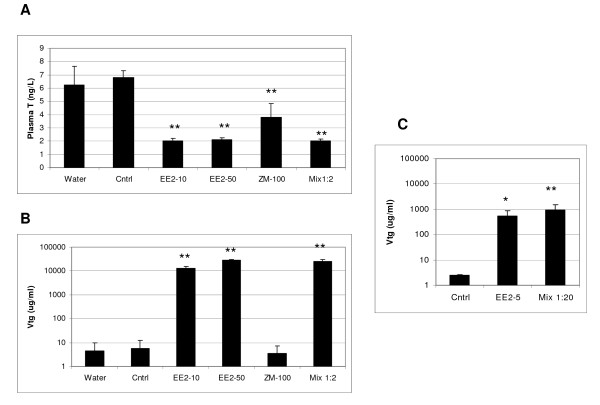
**Phenotypic anchoring measurements for male fathead minnows**. (A and B) Experiment 1. (A) Plasma T concentrations (ng/mL), (B) Plasma vitellogenin concentrations (μg/mL) in the same fish samples. (Cntrl) triethylene glycol control, (EE_2_-10) 10 ng EE_2_/L, (EE_2_-50) 50 ng EE_2_/L, (ZM-100) 100 ng ZM/L, (Mix 1:2) 50 ng EE_2_/L and 100 ng ZM/L. (C) Experiment 2. Plasma VTG concentrations (μg/mL). (EE_2_-5) 5 ng EE_2_/L; (Mix 1:20) 5 ng EE_2_/L and 100 ng ZM/L. Significance ** P ≤ 0.001 and * P ≤ 0.05.

There were no mortalities in any of the treatments. Changes in plasma T and VTG were assessed only for a subset of the exposures for Exp 1 (10 and 50 ng EE_2_/L, 100 ng ZM/L and the mixture of 50 ng EE_2_/L and 100 ng ZM/L) and only plasma VTG was assessed for exposures for Exp 2. Within 48 h, plasma T levels in males were dramatically reduced in all treatments that were measured for Exp 1 (Figure 1A). In the same time frame there was a significant increase in plasma VTG for the two EE_2 _concentrations tested, and for the mixture of 50 ng EE_2_/L and 100 ng ZM/L (Figure 1B). Exposure to 100 ng ZM/L alone did not induce VTG. In the second experiment plasma VTG was significantly up-regulated for the 5 ng EE_2_/L and for the mixture of 5 ng EE_2 _with 100 ng ZM/L (Figure 1C).

### Microarray Results

**Figure 2:**
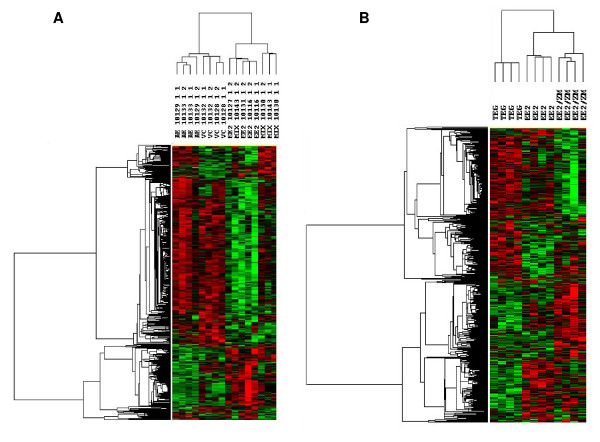
**Bi-directional hierarchical cluster analysis of gene expression changes**. Green indicates down-regulation relative to control and red indicates up-regulation relative to control. Fathead minnows were exposed to (A) 50 ng EE_2_/L (EE_2_), 100 ng ZM/L (ZM), a mixture of 50 ng EE_2_/L and 100 ng ZM/L (Mix), or TEG control (Cntrl). Array analysis was on the 2 K array. (B) 5 ng EE_2_/L (EE_2_), a mixture of 5 ng EE_2_/L with 100 ng ZM/L (EE_2_/ZM) or TEG control (Cntrl). Array analysis was on the 22 K array. Top, clustering was performed by treatment; side, clustering was performed by gene. Each column represents a different array.

As described in the Methods section, two microarray experiments were performed, one using testis from FHM exposed to 50 ng EE_2_/L, 100 ng ZM/L and a combination of both and another using testis from FHM exposed to 5 ng EE_2_/L or to a combination of 5 ng EE_2 _with 100 ng ZM/L. The rest of the samples from other EE_2 _doses were reserved for the quantitative real time PCR (Q-PCR) experiments described below. Samples from the first experiment were analyzed using a 2,000 gene oligonucleotide microarray, and the results are shown in the two sided hierarchical cluster in Figure 2A. The heat map represents genes differentially expressed (*p *< 0.01) between testis of vehicle control and treated fish. We analyzed four biological samples for each of the exposures; each column in Figure 2A represents one of the samples. As expected, control fish clustered together, whereas fish treated with EE_2 _alone or with a combination of EE_2 _and ZM formed a different cluster. Exposure to ZM alone showed the least difference compared to solvent controls; however, even in this comparison there were some differences, suggesting that ZM can influence up- and down-regulation of gene expression in males. There were minor differences between the non-solvent and solvent controls (data not shown).

Exposure to 50 ng EE_2_/L caused many differences in gene expression. The mixture of 100 ng ZM/L and 50 ng EE_2_/L reversed the change for several genes affected by EE_2 _alone, but at this 2:1 ratio the antiestrogen concentration seemed insufficient to totally block the effects of EE_2_.

**Figure 3:**
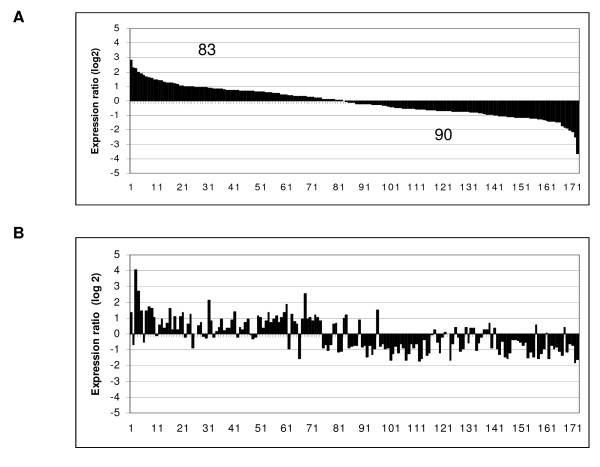
**Comparison of overall gene regulation**. (A) 5 ng EE_2_/L and (B) a mixture of 5 ng EE_2_/L and 100 ng ZM/L as determined by the 22 K array. The genes were ordered according to their expression level in the EE_2 _treatment (*p *< 0.01) and represent median expression values of the four arrays for each condition.

Based on these initial results, we conducted a second study, this time using 5 ng EE_2_/L and a mixture of 100 ng ZM/L with 5 ng EE_2_/L (a ratio of 20:1; Figure 2B). For this analysis, we used a newer 22,000 gene array that had subsequently become available. Exposure to 5 ng EE_2_/L increased plasma VTG (Figure 1C), while the 20-fold excess of ZM in the mixture did not affect this increase. A group of 173 genes was altered (*p *< 0.01) after exposure to either 5 ng EE_2_/L or to the mixture of 5 ng EE_2_/L and 100 ng ZM/L (Figure 3). These changes are plotted in order of their degree of expression change for EE_2 _(Figure 3A), with 83 genes up-regulated and 90 genes down-regulated. Keeping the same order of genes, their fold-expression is plotted for the mixture (Figure 3B). It is clear from this graph that while ZM blocks the EE_2 _effects for some genes, it does not do so for all. There also appears to be a few genes in the middle of this distribution that are significantly altered only by the mixture and not by EE_2 _alone.

**Figure 4:**
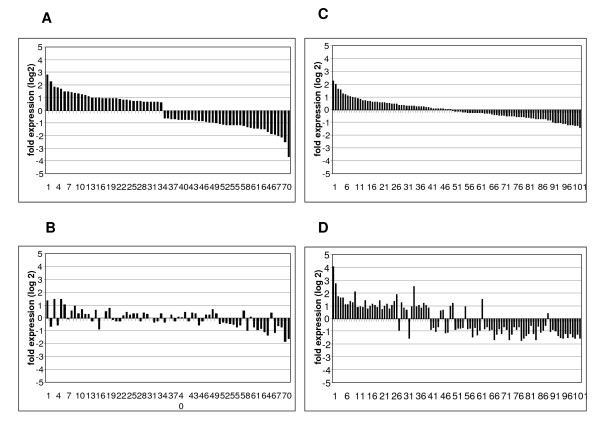
**Competitive and non-competitive blocking of gene expression by ZM**. (A) Genes differentially changed by 5 ng EE_2_/L and (B) blocked by the treatment with the mixture of 5 ng EE_2_/L and 100 ng ZM/L (*p *< 0.01). (C) Genes differentially changed by 5 ng EE_2_/L but (D) whose expression was either not changed or enhanced by the treatment with the mixture of 5 ng EE_2_/L and 100 ng ZM/L. Genes are plotted in order of their change in expression with EE_2 _(*p *< 0.01).

Of the 173 regulated genes, 71 genes were modulated by EE_2 _and blocked by ZM (i.e. reduced expression relative to EE_2 _alone) in the mixture treatment (Figure 4A and 4B). These genes are likely directly regulated by one or more of the soluble ERs and include "cellular processes involved in calcium-dependent cell-cell adhesion," "sugar transporters," "gonadal mesoderm development," "protein repair," and "proteolysis and gas transport" (see Additional file 1). Expression of the remaining 102 genes modulated by EE_2 _was either not affected or was enhanced in either direction by the addition of ZM (Figure 4C and 4D). Many of these genes appear to be involved in signaling cascades, as well as other functions such as "peptide crosslinking," "amino acid biosynthesis and metabolism," "regulation of the immune response," "lipid modification," or "response to stress and to radiation" (see Additional file 2).

### Quantitative real-time reverse transcriptase PCR (Q-PCR)

**Figure 5:**
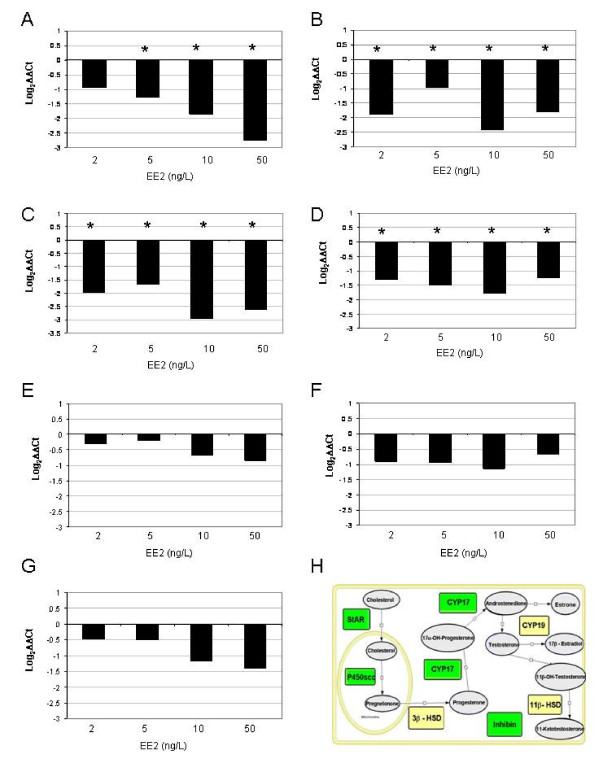
**Q-PCR analysis of mRNAs for proteins involved in steroidogenesis**. Q-PCR results are expressed as fold difference compared to control. Panel A, StAR; Panel B, P450scc; Panel C, CYP17; Panel D, Inhibin; Panel E, CYP19; Panel F, 3β-HSD; Panel G, 11β-HSD; Panel H, model for steroidogenesis. Green boxes refer to mRNAs that are significantly decreased by the treatment in accordance with the Q-PCR graphs illustrated within the panels. Yellow boxes refer to mRNAs that are not significantly changed by the treatments. FHM were treated with TEG, 2, 5, 10 or 50 ng EE_2_/L. StAR, steroidogenic acute regulatory protein, P450scc, Cytochrome P450 side chain cleavage enzyme, CYP17, Cytochrome P450 17, CYP 19, gonadal aromatase, inhibin, hydroxysteroid dehydrogenases including 3β-HSD and 11β-HSD.

Genes that were tested by Q-PCR (Figure 5) were used to both validate the arrays and to focus on genes whose protein products are involved in steroidogenesis and were expected to be affected by EE_2 _[42]. Of the genes tested, steroidogenic acute regulatory protein (StAR), cholesterol side-chain cleavage enzyme (P450scc), cytochrome P450 17α hydroxylase, 17,20 lyase (CYP17) and inhibin were significantly down-regulated by 2 to 50 ng EE_2_/L. Genes for hydroxysteroid dehydrogenases (HSDs) 3β-HSD and 11β-HSD and cytochrome P450 aromatase A-isoform (CYP19A) were not significantly altered, but 11β-HSD and CYP19A showed a downward tendency.

### Functional Analysis

While it is interesting to identify individual genes regulated by EE_2_, most biological processes occur through functional pathways. To assess this, we first assigned as many of the FHM genes as possible to GO categories and to human homologs and then used this information to visualize pathways via Pathway Studio^®^, software from Ariadne Genomics (Rockville, MD, USA). Of the 1,048 genes regulated by any treatment (*p *< 0.05), we were able to assign GO categories to 684 genes (65%). Of these we were able to assign human homologs to 536 genes (51% of the original group).

Because of its environmental significance, we focused on the 5 ng EE_2_/L data for GO analyses. The data set was reduced by statistically determining GO categories for biological processes that were over-represented among the regulated genes which are arranged by increasing *p*-value (up to 0.05) in Table 2. Since GO categories are listed in a hierarchical format, we removed higher order categories if a lower category was present. We found 39 GO biological process categories up-regulated and 51 categories down-regulated. The most significantly up-regulated GO category was "development," while "DNA replication," "response to radiation," "mutagenesis," "DNA repair," "response to light stimulus," "response to DNA damage stimulus," "DNA metabolism," and "response to endogenous stimulus" were the most significantly down-regulated categories.

**Table 2:** GO biological processes that are regulated by 5 ng/L EE_2_

UPREGULATED				
GO ID	GO Biological Process	Fisher p Value	# of Genes Selected	# of Genes on Array
GO:0007275	development	4.35E-03	36	1250
GO:0040036	regulation of fibroblast growth factor receptor signaling	1.58E-02	2	9
GO:0016265	death	1.62E-02	3	27
GO:0018149	peptide cross-linking	1.88E-02	2	10
GO:0050777	negative regulation of immune response	1.88E-02	2	10
GO:0006955	immune response	1.93E-02	10	251
GO:0050776	regulation of immune response	2.09E-02	3	30
GO:0050727	regulation of inflammatory response	2.19E-02	2	11
GO:0006694	steroid biosynthesis	2.26E-02	3	31
GO:0006730	one-carbon compound metabolism	2.44E-02	3	32
GO:0008543	fibroblast growth factor receptor signaling pathway	2.53E-02	2	12
GO:0015758	glucose transport	2.53E-02	2	12
GO:0042770	DNA damage response, signal transduction	2.53E-02	2	12
GO:0008645	hexose transport	2.88E-02	2	13
GO:0015749	monosaccharide transport	2.88E-02	2	13
GO:0008284	positive regulation of cell proliferation	3.24E-02	3	36
GO:0019439	aromatic compound catabolism	3.26E-02	2	14
GO:0007154	cell communication	3.56E-02	41	1692
GO:0043281	regulation of caspase activity	3.65E-02	2	15
GO:0051241	negative regulation of organismal physiological process	3.65E-02	2	15
GO:0009611	response to wounding	3.73E-02	7	167
GO:0009607	response to biotic stimulus	3.91E-02	11	324
GO:0051707	response to other organism	4.03E-02	7	170
GO:0045596	negative regulation of cell differentiation	4.06E-02	2	16
GO:0009605	response to external stimulus	4.18E-02	8	209
GO:0051239	regulation of organismal physiological process	4.19E-02	4	69
GO:0051242	positive regulation of cellular physiological process	4.48E-02	5	103
GO:0006952	defense response	4.48E-02	10	291
GO:0019882	antigen presentation	4.92E-02	2	18
				
DOWNREGULATED				
**GO ID**	**GO Name**	**Fisher p Value**	**# of Genes Selected**	**# of Genes on Array**

GO:0006260	DNA replication	9.27E-04	9	124
GO:0009314	response to radiation	1.51E-03	5	40
GO:0006280	mutagenesis	2.08E-03	2	2
GO:0006281	DNA repair	5.33E-03	9	163
GO:0009416	response to light stimulus	5.51E-03	4	34
GO:0006974	response to DNA damage stimulus	7.91E-03	9	174
GO:0006259	DNA metabolism	8.60E-03	17	458
GO:0009719	response to endogenous stimulus	9.05E-03	9	178
GO:0006139	nucleobase, nucleoside, nucleotide and nucleic acid metabolism	1.29E-02	56	2183
GO:0007623	circadian rhythm	1.74E-02	2	9
GO:0018149	peptide cross-linking	2.07E-02	2	10
GO:0016339	calcium-dependent cell-cell adhesion	2.41E-02	2	11
GO:0006885	regulation of pH	2.78E-02	2	12
GO:0001775	cell activation	3.00E-02	3	33
GO:0045321	immune cell activation	3.00E-02	3	33
GO:0006508	proteolysis	3.17E-02	16	493
GO:0000245	spliceosome assembly	3.17E-02	2	13
GO:0046839	phospholipid dephosphorylation	3.17E-02	2	13
GO:0043283	biopolymer metabolism	3.56E-02	53	2167
GO:0007169	transmembrane receptor protein tyrosine kinase signaling pathway	3.65E-02	5	92
GO:0007156	homophilic cell adhesion	3.94E-02	4	64
GO:0006289	nucleotide-excision repair	4.00E-02	2	15
GO:0009266	response to temperature stimulus	4.00E-02	2	15
GO:0042471	ear morphogenesis	4.00E-02	2	15
GO:0050896	response to stimulus	4.50E-02	26	948
GO:0019941	modification-dependent protein catabolism	4.53E-02	5	98
GO:0043632	modification-dependent macromolecule catabolism	4.53E-02	5	98
GO:0009798	axis specification	4.91E-02	2	17
GO:0030258	lipid modification	4.91E-02	2	17
GO:0042110	T cell activation	4.91E-02	2	17

## Discussion

### Steroidogenesis

We chose three test concentrations of EE_2 _(2, 5, and 10 ng EE_2_/L) with known environmental relevance, and one concentration (50 ng EE_2_/L) higher than normally seen in the environment [43,44]. In our experiments, 10 and 50 ng EE_2_/L decreased plasma T levels, while 5 to 50 ng EE_2_/L increased plasma VTG concentrations in male fish. To our best knowledge, ZM is not present in the environment, although it represents a potentially important mechanism of action, ER antagonism [45]. The concentration we used, 100 ng ZM/L, and the time of exposure, 48 h, are lower and shorter, respectively than in most other studies [46,47] where ZM has been shown to have effects in fish. We chose 100 ng ZM/L to attempt to discern intermediate effects on sensitive genes.

In our study, ZM treatment alone or in the mixture with EE_2 _decreased plasma T levels after 48 h but alone it did not induce plasma VTG concentrations nor did it inhibit the increase in VTG induced by EE_2 _in the mixture in males. In a study by Panter et al [47] ZM significantly decreased VTG after 4 d in E_2_-treated juvenile FHM, but only at a concentration of 76 μg/L, a concentration almost 100-times greater than tested in our experiment. In mummichog (*Fundulus heteroclitus*), there was decrease of plasma T levels in males exposed for 7 days to 250 ng ZM/L but not when treated with 100 ng ZM/L; in those studies there were no effects on VTG levels in males or females with as much as 1,000 ng ZM/L [46].

The Q-PCR data on mRNAs for specific enzymes involved in the biosynthesis of T suggest that the depression of plasma T levels may have occurred directly at the level of steroidogenesis, possibly by direct ER-mediated control of promoters. In the case of CYP17, its down-regulation was blocked by ZM (microarray data), suggesting that it may be regulated via ERs.

### Pathway Analysis

Pathway Studio^® ^[48] was used to visualize changes in gene expression from exposure to 5 ng EE_2_/L, or to the mixture of 5 ng EE_2_/L and a 100 ng ZM/L. This software can be used effectively to compare expression changes with the much larger database of human protein interactions, but only if gene identities are converted to their human homologs. Important caveats for this type of analysis are that there may be many fish genes for which there are no human homologs (e.g., VTG), and some genes in fish belonging to gene families conserved in mammals may actually function differently in fish due to chromosomal duplications. Given these caveats, this type of analysis can help visualize interactions among gene products and their localization in cellular compartments and assist in the formulation of hypotheses that can be tested in future research.

**Figure 6:**
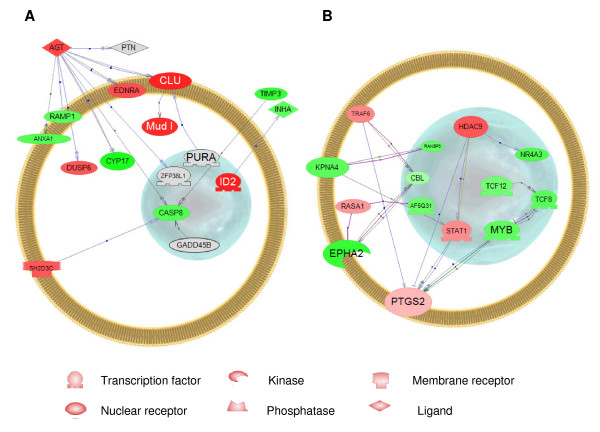
**Pathway Studio analysis**. (A) Competitive interactome consisting of genes that are regulated by 5 ng EE_2_/L and blocked by the mixture of 5 ng EE_2_/L and 100 ng ZM/L. (B) Non-competitive interactome consisting of genes that are regulated by 5 ng EE_2_/L but either not changed or enhanced by the mixture of 5 ng EE_2_/L and 100 ng ZM/L. Red indicates up-regulation, green indicates down-regulation.

An interactome is defined as a set of genes whose protein products are functionally linked together either by direct binding, regulation of activity, regulation of expression, promoter binding, protein modification or molecular transport [48]. Using the databases available in PubMed at NCBI (National Center for Biotechnology Information, ), we have identified interactomes for both the genes that were regulated by EE_2 _and then blocked by the combination of EE_2 _and ZM (Figure 6A – called "competitive interactome" in the discussion below), and those that were regulated by EE_2 _and either not affected by ZM or enhanced (in either direction) by the combination (Figure 6B – called "non-competitive interactome"). We analyzed these separately in order to determine the types of genes that were included in each.

To reduce the complexity of the data, we only examined genes whose products had direct interactions with other entities. We were only able to assign human homologs to about half of the regulated genes, thus our data set underestimates the genes that are directly linked. Entities that showed no linkages to other entities were removed from the figures, but all entities for which we have human homologs are listed in additional files 1 and 2. While it is possible to allow missing entities in the figures in an effort to link all of the entities, this was not attempted because we wanted to exemplify direct interactomes for which there were expression data. Pathway Studio^® ^assigns gene products to cellular compartments depending on their cellular GO terms.

Many of the genes that are found in the "competitive interactome" are known to be regulated by E_2 _and antagonized by estrogen antagonists such as ICI 182,780 in mammalian systems. These genes fit a classical pattern of regulation via soluble ERs. For some of these genes, there is evidence that they contain EREs in their promoters in mammalian systems. For example, angiotensinogen (AGT) is highly prominent in the example shown (Figure 6A) and serves as a node for the "competitive interactome". AGT is normally secreted from the liver into the blood, but there are reports indicating secretion by other organs as well [49]. Our data suggest that it is also produced by the testis of FHM. AGT helps to control blood pressure and, as illustrated by the large number of interactions in Figure 6A, can interact with other proteins in a complex way. In mammals, AGT contains an ERE in its promoter which is up-regulated by both natural and synthetic estrogenic steroids [50]. Thus, the observation that the EE_2_-enhanced expression of this gene is blocked by the EE_2_-ZM mixture lends support to the assumption that the competitive interactome includes genes directly regulated by soluble ERs. The other genes in this interactome have also been implicated in E_2 _signalling in mammalian systems or in cell culture, but there are insufficient data in the literature to determine whether they are all regulated directly by soluble ERs. Furthermore, depending on the tissue the direction of regulation may differ from what we observed in the testis of FHM.

Endothelin receptor type A (EDNRA) is up-regulated by E_2 _during the proliferative phase of the endometrial glandular epithelium [51]. There are no reports on whether or not this up-regulation is through an ERE in the promoter. Different from our results, clusterin (CLU) is down-regulated by E_2 _in rat endometrium, but this regulation is reversed by tamoxifen, another ER antagonist [52]. CLU is known to be regulated by TGF beta and c-fos through an AP-1 site [53]. CLU is a glycoprotein also known as testosterone-repressed prostate message-2 [54]. This gene is expressed in mammalian testis and apparently has many roles including involvement in apoptosis of the seminal vesicle [55]. Complement component factor H (Mud1) contains an imperfect palindrome motif in its promoter in L cells that is present in EREs [56], suggesting that this gene could be directly regulated by soluble ERs. Inhibitor of DNA binding 2 (ID2) has been shown to be down regulated by E_2 _in MCF-7 cells [57] and is directly related to the down-regulation of inhibin alpha, which in turn has a role inhibiting the secretion of FSH from pituitary gonadotrophs [58].

Several genes interconnected with this set were down-regulated by EE_2 _and blocked by ZM in our experiment. For example, receptor (calcitonin) activity modifying protein 1 (RAMP1) has been shown to be down-regulated by E_2 _in rat placenta [59]. Different from our study, some of the down-regulated genes in the FHM testis have been shown to be up-regulated by E_2 _in various mammalian tissues. Pleiotrophin (PTN) is up-regulated in human endometrial epithelial cells [60], and annexin A1 (ANXA1) is up-regulated in a lymphoblastic leukemia cell line [61]. Caspase 8 (CASP8) is regulated by activation of human ERβ but not by ERα [62]. Tissue inhibitor of metalloproteinase 3 (TIMP3) is increased in breast cancer cell growth [63]. It is not clear why the direction of regulation is different in FHM testis, but this may be a tissue specific effect.

In the case of the "non-competitive interactome" (Figure 6B), the genes were differentially expressed in response to EE_2 _exposure and either were not affected by ZM or further amplified by ZM in the mixture. We did not expect to see many genes in this category. This type of effect could be due to activation by E_2 _on non-canonical response elements, as recently demonstrated in transgenic mice expressing a reporter construct containing SP1 sites [33] or by secondary effects that may have occurred in the 48 h timeframe. In Figure 6B, we have accentuated one pathway to illustrate this effect. Prostaglandin-endoperoxide synthase 2 (PTGS2) is a central node in this figure and is induced 1.5 fold by EE_2 _alone but not changed appreciably by the mixture (1.8 fold change). PTGS2 is involved in the synthesis of prostaglandins from arachidonic acid and is influenced by E_2 _in mammalian tissues [64]. Important in this set of genes is the gene for "signal transducer and activator of transcription 1" (STAT1) which is a critical transcription factor involved in the JAK-STAT signalling pathway central for innate immunity [65] and apoptosis [66], among other functions. This transcription factor is also activated via the retinoic acid receptor signalling pathway [67], thus bridging both the E_2 _and retinoic acid pathways. Also important is histone deacetylase 9 (HDAC9), a gene product that is involved in chromatin remodelling, allowing access of transcription factors to regions in DNA. No information exists regarding the influence of estrogen on HDAC9 in mammalian tissues but inactivation of other histone deacetlyases is an important step for ER activation in cell lines no longer responsive to E_2 _[68]. Tnf receptor-associated factor 6 (TRAF6) is a protein known to be involved in signal transduction through membrane receptors [69], and RAS p21 protein activator 1 appears to play a role in Ras GTPase mediated signal transduction [70].

Among the down-regulated genes were several transcription factors, including transcription factor 12 (TCF12) involved in control of immunoglobulin transcription [71], transcription factor 8 (TCF8), a negative regulator of cadherin [72], myeloblastosis oncogene (MYB), known to be involved in estrogen signalling in some breast cancer cells [73] and AF4/FMR2 family, member 4 (AF5Q31), which functions as a transcriptional regulator in testicular somatic cells, essential for male germ cell differentiation and survival [74]. Karyopherin (importin) alpha 4 (KPNA4) helps import proteins into the nucleus during spermatogenesis [75]. RAN binding protein 3 isoform b (RANBP3) links the RAS and PI3-kinase signaling pathways with nuclear transport [76] and Eph receptor A2 (EPHA2), is a protein in the tyrosine kinase family [77]. The roles of other genes in this interactome are listed in additional file 2.

It is not clear at this time why so many genes have escaped antagonism by ZM in the mixture exposure. It is possible that they are activated via non-canonical ER interactions with other transcription factors [33] or are the results of activation through G protein-coupled receptor 30 (GPR30), a membrane-bound estrogen receptor [35]. A large percentage of genes in the non-competitive interactome function in non-genomic signaling pathways, raising the possibility that these genes are all regulated via membrane receptors which escape antagonism. Further work will be required to sort out exactly how each of these genes is regulated.

## Conclusion

We used genomics to try to elucidate the mechanisms of action of estrogenic and anti-estrogenic compounds and their potential effects on aquatic organisms. Our data provides some insight into the estrogen-regulated effects, suggesting that response to estrogens occurs via different mechanisms. The use of an estrogen/antiestrogen mixture provides a distinction among different modes of action of estrogenic compounds: through canonical binding to soluble ERs; membrane ERs; or some other potential mechanisms that may not be blocked by pure antiestrogens.

## Methods

### Fish Exposure and Tissue Collection

Reproductively-mature, pond-reared FHM were purchased from Andersen Minnow Farm, AR, 4 days prior to starting the experiment. Upon arrival, the fish were treated for parasites and bacteria by a prophylactic salt-water dip (3%, 1 min). Males were separated from the population the following day, and acclimated in the treatment aquaria for 48 h. The water used for this study was carbon-filtered, dechlorinated tap water.

The exposure system consisted of 40 L glass aquaria. Each exposure was conducted in quadruplicate and each aquarium contained eight male FHM in 25 L of treatment water. Test chemicals for each treatment group (100 L for 4 aquaria) were prepared in separate (by treatment) 250 L fiberglass tanks the day of exposure. Aquaria were equilibrated with test chemicals for 24 h prior to the introduction of fish. Test solutions were renewed to 90% of the 25 L exposure volume after 24 h and the exposure was ended at 48 h. The positions of the treatment tanks were randomized and test initiation times were staggered to ensure an exposure/sampling interval of 48 h. The fish were not fed the day before and during the experiment. Temperature was maintained at 25°C with a photoperiod of 16 h light: 8 h dark.

### Exposure Solutions

EE_2 _was purchased from Sigma Chemical Company (St. Louis, MO). ZM189,154 was a generous gift from AstraZeneca. Working solutions for each test chemical consisted of 1 mg/ml test compound in 70% triethylene glycol (TEG) and 28.5% ethanol. This working solution was further diluted to make stock solutions for each treatment (nominal concentrations of 2, 5, 10 and 50 ng EE_2_/L and 100 ng ZM/L and mixtures containing 5 ng EE_2_/L or 50 ng EE_2_/L and 100 ng ZM/L), so as to maintain a concentration of 50 μl TEG/L of test water. EE_2 _concentrations spanned the environmentally relevant levels (2–10 ng EE_2_/L) to a concentration higher than would typically occur in the environment. The antiestrogen ZM concentration was chosen to be higher than EE_2 _so that it could effectively block action on the ERs. The concentration of ZM in the two mixtures was 100 ng ZM/L.

Water samples were collected at the start of the exposures (0 hr), and after 24 h (both before and after change of tank water) and after 48 h. A sample of the test solution (1 L) was collected in an amber glass bottle with a teflon cap and stored at 4°C. The water was passed through an AccuBond II ODS-C18 solid phase extraction column (Agilent, Palo Alto, CA) and the EE_2 _was eluted with 5 ml dichloromethane. After drying, the EE_2_residue was reconstituted in 1 ml of buffer and analyzed using an enzyme-linked immunosorbent assay (ELISA) kit (Abraxis, Los Angeles, CA), following the manufacturer's instructions. The detection limit for this assay is 50 ng EE_2_/L in the reconstituted solution. We were not able to determine actual concentrations of ZM, so we report only nominal concentrations.

All procedures involving live fish were reviewed and approved by the University of Florida Institutional Animal Care and Use Committee (IACUC). At the conclusion of the exposures, fish were anesthetized (MS-222), weighed to the nearest 0.1 g and blood samples were collected from the caudal vasculature for analysis of VTG and T concentrations, as described below. The testes were removed and cut into small pieces. Dissected tissues were flash frozen using liquid nitrogen and stored at -80°C until needed.

### Vitellogenin Assay

Plasma concentrations of VTG were determined by ELISA using a monoclonal antibody, 2D3, previously validated for the FHM [78]. The limit of detection for the FHM VTG ELISA in plasma was 0.5 μg/mL. All assays were performed in triplicate and reported as the mean of the three measurements. The coefficient of variation was < 10% for all samples analyzed. Inter and intra-assay variability was routinely measured by analyzing positive controls on several plates and found to be < 10% and < 5%, respectively.

### Testosterone Radioimmunoassay

Plasma concentrations of T were measured using a radioimmunoassay (RIA) validated for the FHM based on a slight modification of a previously published protocol [79]. The antibody against T, 20-TR05, was purchased from Fitzgerald Industries International, Concord, MA. Tritiated label ([1,2,6,7-^3^H] T) was from GE Healthcare (Piscataway, NJ). The T standard (Sigma T-1500) was obtained from Sigma Chemical Company (St. Louis, MO). Plasma samples (12 μL each) were extracted with 2 mL of ethyl ether, as described previously [79]. The extraction efficiency was 93%. Samples were analyzed in duplicate. The intraassay coefficients of variance were generally ≤ 5% and all samples were run in one assay to prevent interassay variability.

### Data analysis

Plasma concentrations of T were analyzed by one-way ANOVA, followed by Fisher Protected Least Significant Difference (PLSD) test for post-hoc analysis. All analyses were carried out using StatView 5.0 (SAS Institute, Inc., Cary, NC). Homoscedasticity was assessed using F – tests, and, where necessary (p < 0.05), data were log transformed [80]. All data are reported as nontransformed values, as mean ± SEM, and significance was determined at *p*-value < 0.05. Plasma VTG concentrations were analyzed by Dunnett's pairwise multiple comparisons on log transformed data.

### RNA Extraction

Total RNA was isolated from 30–50 mg FHM gonadal tissue with the RNA Stat-60 reagent (Tel-test, Friendswood, TX), as previously described [81]. Total RNA was treated with DNase and the quality assessed with an Agilent 2100 BioAnalyzer (Agilent, Palo Alto, CA), and the quantity determined on a NanoDrop spectrophotometer (NanoDrop Technologies, Wilmington, DE). RNA was stored at -80°C until further use.

### Microarrays

Fathead minnow microarrays manufactured by Agilent (Palo Alto, CA) were purchased from EcoArray (Alachua, FL). For the first experiment we used a targeted 2,000 gene array (GPL6516) while for the other we employed a 22,000 gene array (4 × 44 K format, GPL7282). Array hybridizations were performed using a reference design. The reference material, which was used for all studies, consisted of equal amounts of RNA from both female and male tissues (liver, brain and gonad). Four replicates consisting of four different individuals were analyzed for each of the treatments (solvent (TEG) control, non-solvent control, EE_2_, ZM, EE_2_/ZM). The cDNA synthesis, cRNA labeling and hybridization were performed following the manufacturer's kits and protocols (Agilent Low RNA Input Fluorescent Linear Amplification Kit and Agilent 60-mer oligo microarray processing protocol; Agilent, Palo Alto, CA). The gonad samples were labeled with Cy_5 _while the reference sample was labeled with Cy_3_. Once the labeling was complete, samples were hybridized to the microarray using conditions recommended by the manufacturer. After hybridizing for 17 h, microarrays were washed and then scanned with a laser-based detection system (Agilent, Palo Alto, CA). Text versions of the Agilent raw data have been deposited at the Gene Expression Omnibus website (GEO: ; Accession series record number GSE14235).

### Bioinformatics

Microarray image processing and data pre-processing were performed using Agilent's Feature Extraction software v 9.5 (Agilent, 2007). The intensity of each spot was summarized by the median pixel intensity. A log_2 _transformed signal ratio between the experimental channel and the reference channel was calculated for each spot, followed by within-array LOWESS transformation and between array scale normalization on median intensities [82].

Two-way ANOVA was performed on log_2 _transformed signal ratios of each probe individually, followed by Tukey-HSD pair-wise comparisons to determine genes whose expression was significantly regulated by the treatments. A *p*-value ≤ 0.05 was used as the cutoff. Genes whose fold expression changes were less than 1.5 fold were eliminated from further analyses irrespective of statistical significance.

GeneOntology (GO) annotations were provided by EcoArray Inc. based largely on homologies between FHM genes and human genes. Overrepresentation of differentially expressed genes in the biological process GO category was determined by Fisher Exact Test with a *p*-value ≤ 0.05 as a cutoff, and the false discovery rate was determined [83]. PathwayStudio^® ^software [48] from Ariadne Genomics (Rockville, MD) was used to determine the list of common regulators among the genes that were differentially expressed in the experiments.

### Real-time Polymerase Chain Reaction (Q-PCR)

Total RNA (1 μg) was reverse transcribed into cDNA using 3 μl random primers (0.1 μg/μl), 0.8 μl dNTP mix (25 mM each dNTP), 2 μl transcription buffer (10×), 1 μl StrataScript RT (50 U/μl), and 0.5 μl RNAse Block (40 U/μl) in a final volume of 20 μl (all reverse transcription reagents were from Stratagene, La Jolla, CA). The resulting cDNA was used as a template for Q-PCR. Specific primers for selected genes were designed to perform Q-PCR (Table 3).

**Table 3:** Real time PCR primers

**GENE**	**FWD PRIMER (5' – 3')**	**REV PRIMER (5' – 3')**
CYP 17	ACACAAGGTGGATTACAGTGATAACGT	CTGCGTTTGGCCCTCAGA
3β-HSD	ATGAGATGCCCTACCCAAAGAC	CCCTTTACCTTTGTGCCATTG
11β-HSD	GCATCGGCGAGCAGTTG	CTCCTCGCCGTGATAACGA
INHB	ACCACGCTACTCGGGATCAA	CGGAGGGACTTCATGCTCTCT
CYP19A	TGCTGACACATGCAGAAAAACTC	CAGCTCTCCGTGGCTCTGA
P450scc	CGACACCCGGACTTGCA	CACGTCTCCTTTAGAGGTGATACG
StAR	CTTGAACAGCAAACAGATGACCTT	CTCCCCCATTTGTTCCATGT

Each Q-PCR reaction consisted of 1× iQ SYBR Green Supermix (Bio-Rad, Hercules, CA), 0.4 μM primers and 1 μl of cDNA in a 25 μl reaction. The Q-PCR conditions were 95°C for 3 min and 40 cycles at 95°C for 15 sec and 60°C for 1 min in an iCycler Thermal Cycler (Bio-Rad, Hercules, CA). The Q-PCR results were normalized to 18S rRNA (Applied Biosystems, Foster City, CA) and analyzed using the ΔΔCt method, compared to the vehicle controls. We measured the following mRNAs: cytochrome P450 17α hydroxylase, 17,20 lyase (CYP17) [84], steroidogenic acute regulatory protein (StAR) [85], cholesterol side-chain cleavage enzyme (P450scc) [85], hydroxysteroid dehydrogenases (HSDs) 3β-HSD [85] and 11β-HSD [86], inhibin (INHB) and cytochrome P450 aromatase A-isoform (CYP19A) [87].

## List of abbreviations

AF5Q31: AF4/FMR2 family, member 4; AGT: angiotensinogen; ANXA1: annexin A1; AP-1: activating protein 1; 3β-HSD: 3 beta hydroxysteroid dehydrogenase; 11β-HSD: 11 beta hydroxysteroid dehydrogenase; CASP8: caspase 8; CBL: CBL E3 ubiquitin protein ligase; CLU: clusterin; Cntrl: control; CYP 17: cytochrome P450, family 17, subfamily a, polypeptide 1; CYP19A: cytochrome P450 aromatase A-isoform; DUSP6: dual specific phosphatase 6; EDCs: Endocrine disrupting chemicals; E_2_: 17β estradiol; EE_2_: 17α ethinylestradiol; ELISA: enzyme-linked immunosorbent assay; ENDRA: endothelin receptor type A; EPHA2: Eph receptor A2; ER: estrogen receptor; FHM: fathead minnow; GADD45B: growth arrest and DNA-damage-inducible 45 beta; GO: gene ontology; HDAC9: histone deacetylase 9; ID2: inhibitor of DNA binding 2; INHA: inhibin alpha; KPNA4: karyopherin (importin) alpha 4; MYB: myeloblastosis oncogene; Mud 1: complement component factor H; NR4A3: nuclear receptor subfamily 4, group A, member 3; Q-PCR: quantitative real time reverse transcriptase polymerase chain reaction; P450scc: Cytochrome P450 side chain cleavage enzyme; PTGS2: prostaglandin-endoperoxide synthase 2; PTN: pleiotrophin; PURA: purine rich element binding protein A; RAMP1: receptor (calcitonin) activity modifying protein 1; RANBP3: RAN binding protein 3 isoform; RASA1: RAS p21 protein activator 1; RIA: radioimmunoassay; SH2D3C: SH2 domain containing 3C; Sp1: stimulatory protein 1; StAR: steroidogenic acute regulatory protein; STAT1: signal transducer and activator of transcription 1; T: testosterone; TCF8: transcription factor 8; TCF12: transcription factor 12; TIMP3: tissue inhibitor of metalloproteinase 3; TRAF6: Tnf receptor-associated factor 6; VTG: vitellogenin; ZFP36L1: zinc finger protein 36, C3H type-like 1; ZM: ZM 189,154.

## Authors' contributions

NGR, NDD, KHW, MSS and EFO, conceived of the study and helped draft the manuscript; NGR, DLV, EFO, and KJK, performed the experimental work; LL, performed bioinformatics analysis; EJP, GTA, revised the manuscript critically for important intellectual content. All authors read and approved the final manuscript.

## Supplementary Material

Additional file 1 **Human homologs of genes competitively regulated by EE_2 _and ZM** Click here for file

Additional file 2 **Human homologs of genes non-competitively regulated by EE_2 _and ZM** Click here for file
